# Complete chloroplast genome structure of four *Ulmus* species and *Hemiptelea davidii* and comparative analysis within Ulmaceae species

**DOI:** 10.1038/s41598-022-20184-w

**Published:** 2022-09-24

**Authors:** Yichao Liu, Yongtan Li, Shuxiang Feng, Shufang Yan, Jinmao Wang, Yinran Huang, Minsheng Yang

**Affiliations:** 1 Institute of Forest Biotechnology, Forestry College, Hebei Agricultural University, Baoding, 071000 China; 2 Hebei Key Laboratory for Tree Genetic Resources and Forest Protection, Baoding, 071000 China; 3 Hebei Forestry and Grassland Science Research Institute, Shijiazhuang, 050000 China; 4 Hebei Forest City Constructed Technology Innovation Center, Shijiazhuang, 050000 China

**Keywords:** Evolution, Genetics, Molecular biology, Plant sciences, Structural biology

## Abstract

In this study, the chloroplast (cp) genomes of *Hemiptelea davidii*, *Ulmus parvifolia*, *Ulmus lamellosa*, *Ulmus castaneifolia*, and *Ulmus pumila* ‘zhonghuajinye’ were spliced, assembled and annotated using the Illumina HiSeq PE150 sequencing platform, and then compared to the cp genomes of other *Ulmus* and Ulmaceae species. The results indicated that the cp genomes of the five sequenced species showed a typical tetrad structure with full lengths ranging from 159,113 to 160,388 bp. The large single copy (LSC), inverted repeat (IR), and small single copy (SSC) lengths were in the range of 87,736–88,466 bp, 26,317–26,622 bp and 18,485–19,024 bp, respectively. A total of 130–131 genes were annotated, including 85–86 protein-coding genes, 37 tRNA genes and eight rRNA genes. The GC contents of the five species were similar, ranging from 35.30 to 35.62%. Besides, the GC content was different in different region and the GC content in IR region was the highest. A total of 64-133 single sequence repeat (SSR) loci were identified among all 21 Ulmaceae species. The (A)_n_ and (T)_n_ types of mononucleotide were highest in number, and the lengths were primarily distributed in 10–12 bp, with a clear AT preference. A branch-site model and a Bayes Empirical Bayes analysis indicated that the *rps15* and *rbcL* had the positive selection sites. Besides, the analysis of mVISTA and sliding windows got a lot of hotspots such as *trnH/psbA*, *rps16/trnQ*, *trnS/trnG*, *trnG/trnR* and *rpl32/trnL*, which could be utilized as potential markers for the species identification and phylogeny reconstruction within *Ulmus* in the further studies. Moreover, the evolutionary tree of Ulmaceae species based on common protein genes, whole cp genome sequences and common genes in IR region of the 23 Ulmaceae species were constructed using the ML method. The results showed that these Ulmaceae species were divided into two branches, one that included *Ulmus*, *Zelkova* and *Hemiptelea*, among which *Hemiptelea* was the first to differentiate and one that included *Celtis*, *Trema*, *Pteroceltis*, *Gironniera* and *Aphananthe*. Besides, these variations found in this study could be used for the classification, identification and phylogenetic study of *Ulmus* species. Our study provided important genetic information to support further investigations into the phylogenetic development and adaptive evolution of *Ulmus* and Ulmaceae species.

## Introduction

Ulmaceae includes approximately 16 genera and 230 species that are primarily distributed in the tropical-to-cold temperate zone of the Northern Hemisphere. Currently, eight genera of Ulmaceae are found in China, including *Ulmus*, *Celtis*, *Aphananthe*, *Trema*, *Gironniera*, *Zelkova*, *Hemiptelea*, and *Pteroceltis*. These genera include 46 species and ten varieties^1^ distributed throughout the country, and *Ulmus* accounts for nearly half of these species. Elms generally exhibit extensive adaptability and strong resistance^2,3^, mainly in afforestation and landscape greening applications^4,5^. In addition, most types of elm woods are hard, delicate, wear-resistant, tough, and excellent in quality, and can be used for furniture, construction, and bridges^6^. Numerous beneficial substances can be found in the bark and root bark of elms, many of which have high medicinal value^7,8^. The phloem of elm has high viscosity and can be used as a natural plant adhesive, and the leaves can be used as animal feed^9^. In addition, the seed oils of *Gironniera*, *Ulmus*, *Aphananthe*, and *Celtis* can be used for industrial purposes^10^.

Plant palynological fossils and other studies have documented that elms have existed since approximately the third century of the geological age^11,12^. As an ancient Tertiary tree family, Ulmaceae is rich in germplasm resources. The large numbers of naturally occurring polyploids and mutants^13,14^ and interspecific and intraspecific hybrids^15^ lend themselves to extensive elm varieties worldwide, with complex genetic backgrounds^16–18^. However, because previous plant classification and identification methods focused on morphological characteristics, pollen characteristics, and flavonoid differential substances^19^ but generally lacked molecular identification. Many differences and controversies exist in the evolution and classification of Ulmaceae plants^20–24^, including the attribution of *Ulmus*, *Pteroceltis*, *Gironniera*, *Trema*, and *Aphananthe*^25,26^, and the classification and species determination of *Ulmus* vary widely^27,28^.

In this study, we sequenced, assembled and annotated the cp genomes of *U. parvifolia*, *H. davidii*, *U. lamellosa*, *U. castaneifolia* and *U. pumila* ‘zhonghuajinye’, and compared their sequences with related species. Moreover, this present study using the cp genome to construct the evolutionary tree aimed to improve our understanding of evolution within Ulmaceae species. The plant-specific cp genome is relatively independent of the nuclear genome. Compared to nuclear genome sequences, the cp genome exhibits a low molecular weight, low nucleotide substitution rate and slow structural variation; therefore, it is increasingly used to solve deep phylogenetic problems within plants^29–31^. Besides, the structural characteristics and variation of the cp genomes of *Ulmus* and Ulmaceae species were preliminarily documented to obtain comprehensive understanding the structure of plastomes within Ulmaceae, which will help to lay the foundation for the accurate identification of *Ulmus* and Ulmaceae species classification and genome evolution.

## Materials and methods

### Test materials

**Figure 1:**
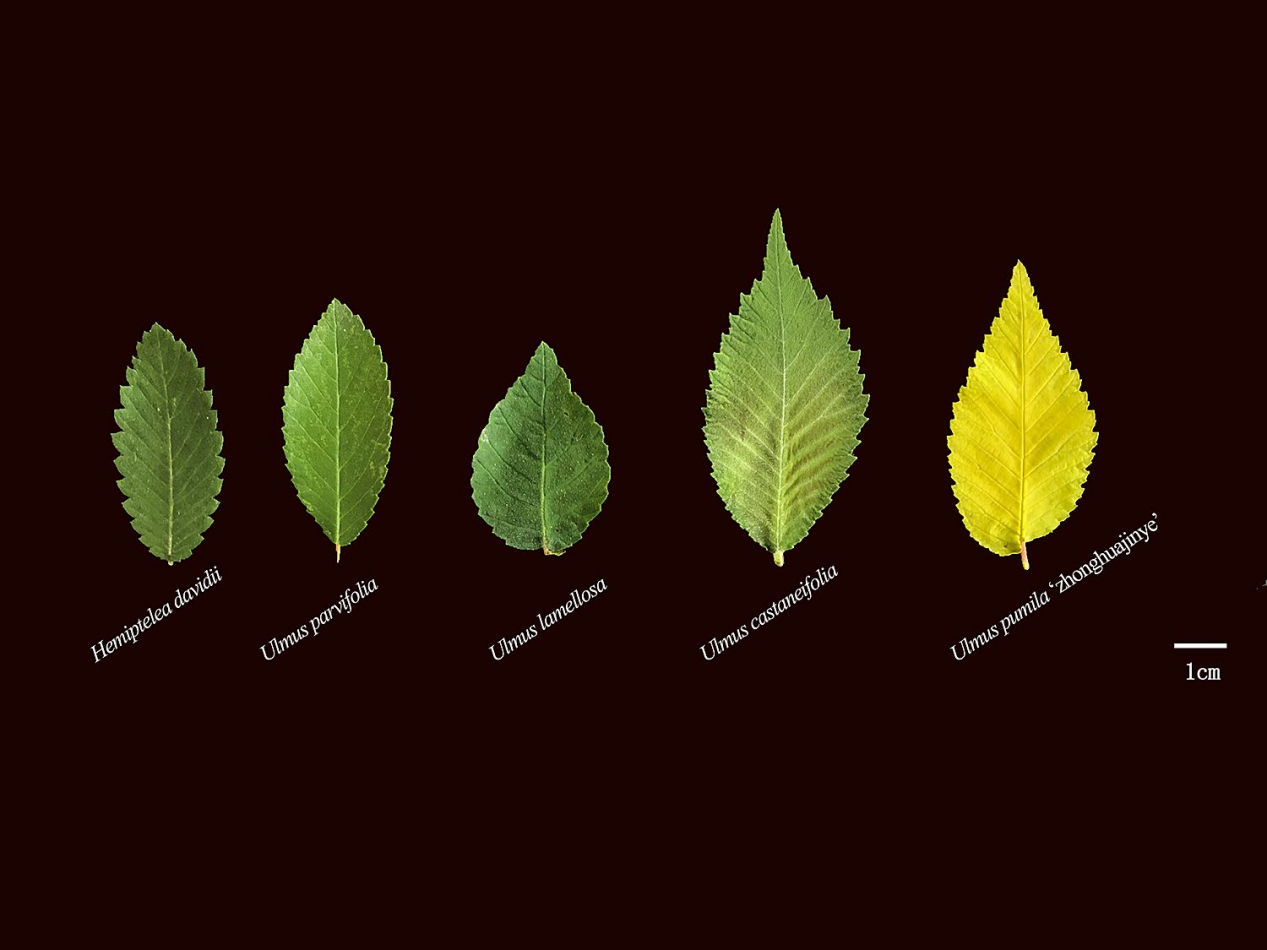
Leaves characteristic of four *Ulmus* species and *H. davidii.*

*Hemiptelea davidii*, *Ulmus parvifolia*, *Ulmus lamellosa*, *Ulmus castaneifolia* and *Ulmus pumila* ‘zhonghuajinye’ (Fig. 1) were used as the focal experimental species. In May 2019, young and healthy mature leaves on annual branches of each sample were selected from the Germplasm Resources Nursery of the Hebei Forestry and Grassland Science Research Institute. All methods were carried out in accordance with relevant guidelines and regulations.

### DNA extraction and Illumina sequencing

The leaves were cleaned with ultrapure water and then immediately placed into liquid nitrogen and stored at − 80 °C. A plant DNA extraction kit (TIANGEN Biotech, Beijing, China) was used to extract the total DNA from fresh young leaves of each sample. The integrity and quality of total DNA were detected using agarose gel and a NanoDrop2000 microspectrophotometer (Thermo Fisher Scientific, Waltham, MA, USA). The qualified samples were sent to Beijing Zhongxing Bomai Technology Co., Ltd. (Beijing, China) for cp genome sequencing using the Illumina HiSeq PE150 double-end sequencing strategy.

### Chloroplast genome assembly, annotation and visualization

Clean reads were filtered using Trimmomatic ver. 0.33 software^32^ to acquire clean reads by deleting adaptors and low quality reads. GetOrganelle^33^ was used to assemble cp genome sequences, which were then annotated using GeSeq software^34^. HMMER and ARAGORN v1.2.38^35^ were used to ensure the accuracy of the predictions for the encoded protein and RNA genes, respectively. Moreover, the Chloroplotc^36^ was used to draw the cp genome maps. Finally, the newly obtained cp genomes were uploaded to the NCBI database.

### Sequence and genome comparison analyses

The single sequence repeats (SSRs) were determined using MISA^37^ among the cp genomes of 23 Ulmaceae species. The parameter settings for single mononucleotide, dinucleotide, trinucleotide, tetranucleotide, pentanucleotide and hexanucleotide repeats were ten, six, five, five, five and five, respectively. REPuter^38^ was to identify and locate the repeat sequences among Ulmaceae species including forward repeats (F), reverse repeats (R), palindromic repeats (P) and complement repeats (C) and the following parameters were used: (1) 30 bp minimum repeat size and (2) 90% or greater sequence identity (Hamming distance = 3). Tandem Repeats Finder ver. 4.04^39^ was used to analyze and detect tandem repeats, with the default parameters. The mVISTA software^40^ (Frazer et al., 2004) was used to examine the genetic divergence among Ulmaceae species using *U. pumila* as reference, in the LAGAN model. We also conducted a window analysis to identify the nucleotide diversity (Pi) among the cp genomes of 21 Ulmaceae species using DnaSP v5.10 software^41^.

### Ka/Ks and positive selection on plastid genes

A total of 77 protein coding genes from 23 cp genomes of Ulmaceae species were selected for positive selected genes (PSGs) identification and analysis. First, MAFFT v7^42^ was used to compare the amino acid sequences of each gene. PhyML v3.0 software^43^ was then used to construct the phylogenetic tree based on the maximum likelihood (ML) method for the above multiple-sequence alignment results. Subsequently, trimAl v1.4^44^ was used for trimming, and PAML v4.9 CodeML was used for branch-site analysis. The parameters of Model A and Model A null in branch site were Model A (Model = 2, NSsites = 2, fix/omega = 0, omega = 2) and Model A null (Model = 2, NSsites = 2, fix/omega = 1, omega = 1). The likelihood ratio test (LRT) of paml chi2 (chi2 d.f.2ΔlnL) was used to obtain the LRT *P* value. False discovery rate correction was performed on the LRT *P* value. Gene with *P * value < 0.05 was selected as PSG. Lastly, the posterior probabilities of amino acid sites were calculated using Bayes Empirical Bayes (BEB) to determine whether the sites were positively selected.

### Phylogenetic analyses

23 Ulmaceae species were selected from the NCBI database (Table S1). The phylogenetic trees were constructed with *Arabidopsis thaliana* as an outgroup. The cluster analyses were conducted based on the whole cp genome sequence, common protein genes (*accD*, *atpA*, *atpB*, *atpE*, *atpF*, *atpH*, *atpI*, *ccsA*, *cemA*, *clpP*, *matK*, *ndhA*, *ndhB*, *ndhD*, *ndhE*, *ndhF*, *ndhG*, *ndhH*, *ndhI*, *ndhJ*, *ndhK*, *petA*, *petB*, *petD*, *petG*, *petL*, *petN*, *psaA*, *psaB*, *psaC*, *psaI*, *psaJ*, *psbA*, *psbB*, *psbC*, *psbD*, *psbE*, *psbF*, *psbH*, *psbI*, *psbJ*, *psbK*, *psbL*, *psbM*, *psbN*, *psbT*, *psbZ*, *rbcL*, *rpl14*, *rpl16*, *rpl20*, *rpl22*, *rpl23*, *rpl2*, *rpl32*, *rpl33*, *rpl36*, *rpoA*, *rpoB*, *rpoC1*, *rpoC2*, *rps11*, *rps12*, *rps14*, *rps15*, *rps16*, *rps18*, *rps19*, *rps2*, *rps3*, *rps4*, *rps7*, *rps8*, *ycf1*, *ycf2*, *ycf3* and *ycf4*) and common genes in IR region (*ndhB*, *rpl2*, *rpl23*, *rps12*, *rps7*, *ycf1* and *ycf2*). MAFFT v7 was used to align the cpDNAs sequences under default parameters^42^, and the alignment was trimmed by Gblocks/0.91b to remove low-quality regions with the parameters: − t = d − b4 = 5 − b5 = h^45^. The Maximum-likelihood (ML) method was performed for the phylogenetic analyses using PhyML v3.0^43^. Nucleotide substitution model selection was estimated with jModelTest 2.1.10^46^. The model GTR + I + G was selected for ML analyses with 1,000 bootstrap replicates to calculate the bootstrap values (BS) of the topology. Moreover, the results were treated with iTOL 3.4.3^47^.

## Results and analysis

### Chloroplast characteristics of *Ulmus* species

**Figure 2:**
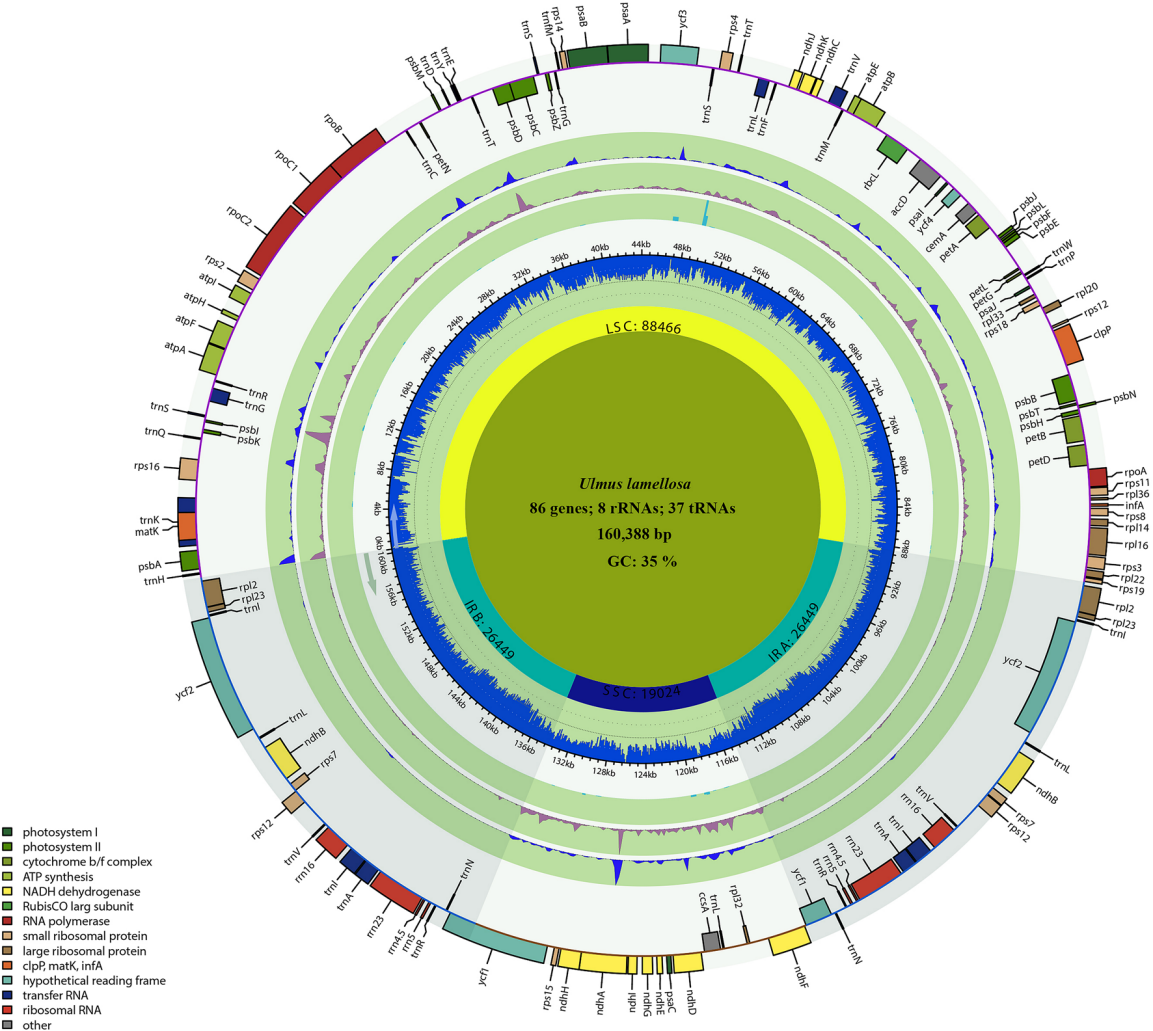
The cp genome maps of *Ulmus* species*.* The species name and specific information regarding the genome (length, GC content, and the number of genes) are depicted in the center of the plot. Extending outward, the four layers are the nucleotide diversity of *U. pumila* ‘zhonghuajinye’, *U. parvifolia, U. castaneifolia* and *U. lamellosa* respectively.

In the present study, the cp genomes of *H. davidii*, *U. parvifolia*, *U. lamellosa*, *U. castaneifolia* and *U. pumila* ‘zhonghuajinye’ were sequenced, assembled and annotated. As shown in Fig. 2 and Table 1, the cp genomes of the five species were covalently closed double-chain cyclic molecules with a typical four-segment structure, and the sizes ranged from 159,113 to 160,388 bp (Table 1). *U. lamellose* had the largest genome, while *U. pumila* ‘zhonghuajinye’ had the smallest. The lengths of the LSC in each segment varied greatly (87,736–88,466 bp), with a difference of 730 bp. The longest LSC occurred in *U. lamellosa*, followed by *U. castaneifolia*, *U. pumila* ‘zhonghuajinye’, *H. davidii*, and *U. parvifolia*. The lengths of the SSC region ranged from 18,485 to 19,024 bp, with a difference of 539 bp. And the variation range of the SSC region was smaller than that of the LSC region. Among them, *U. lamellosa* had the largest SSC region and *U. pumila* ‘zhonghuajinye’ had the smallest. Besides, the smallest IR region occurred in *U. pumila* ‘zhonghuajinye’ (26,317 bp), while the largest was found in *H. davidii* (26,622 bp). The cp genome of *H. davidii* with a total of 130 genes contained the smallest number of genes of the five species, while the other four species had 131 genes each. The five species contained 85–86 protein-coding genes, 37 tRNAs and eight rRNAs. In addition, the coding region was longer than the non-coding region and the coding region (36.62–36.74%) had significantly higher GC content than the non-coding region (33.96–34.48%). Moreover, the GC content in rRNA was higher than that in tRNA.

**Table 1:** The basic characteristics of the cp genomes of four *Ulmus* species and *H. davidii.*

	*Hemiptelea davidii*	*Ulmus parvifolia*	*Ulmus lamellosa*	*Ulmus pumila* ‘zhonghuajinye’	*Ulmus castaneifolia*
Total length (bp)	159,803	159,199	160,388	159,113	159,925
Total gene number	130	131	131	131	131
Total GC(%)	35.30	35.60	35.43	35.62	35.49
LSC length (bp)	87,798	87,736	88,466	87,994	88,154
GC (%)	32.74	33.08	32.90	33.08	32.96
SSC length (bp)	18,761	18,743	19,024	18,485	18,955
GC (%)	28.08	28.60	28.15	28.63	28.34
IR length (bp)	26,622	26,360	26,449	26,317	26,408
GC (%)	42.07	42.30	42.25	42.34	42.28
Coding region length	80,517	80,472	80,532	80,559	80,487
Coding region GC (%)	36.62	36.73	36.74	36.73	36.72
Noncoding region length (bp)	79,286	78,727	79,856	78,554	79,438
Noncoding region GC (%)	33.96	34.44	34.11	34.48	34.24
Protein-coding gene number	85	86	86	86	86
tRNA	37	37	37	37	37
tRNA GC (%)	53.12	53.12	53.12	53.12	53.12
rRNA	8	8	8	8	8
rRNA GC (%)	55.29	55.33	55.33	55.33	55.33

**Figure 3:**
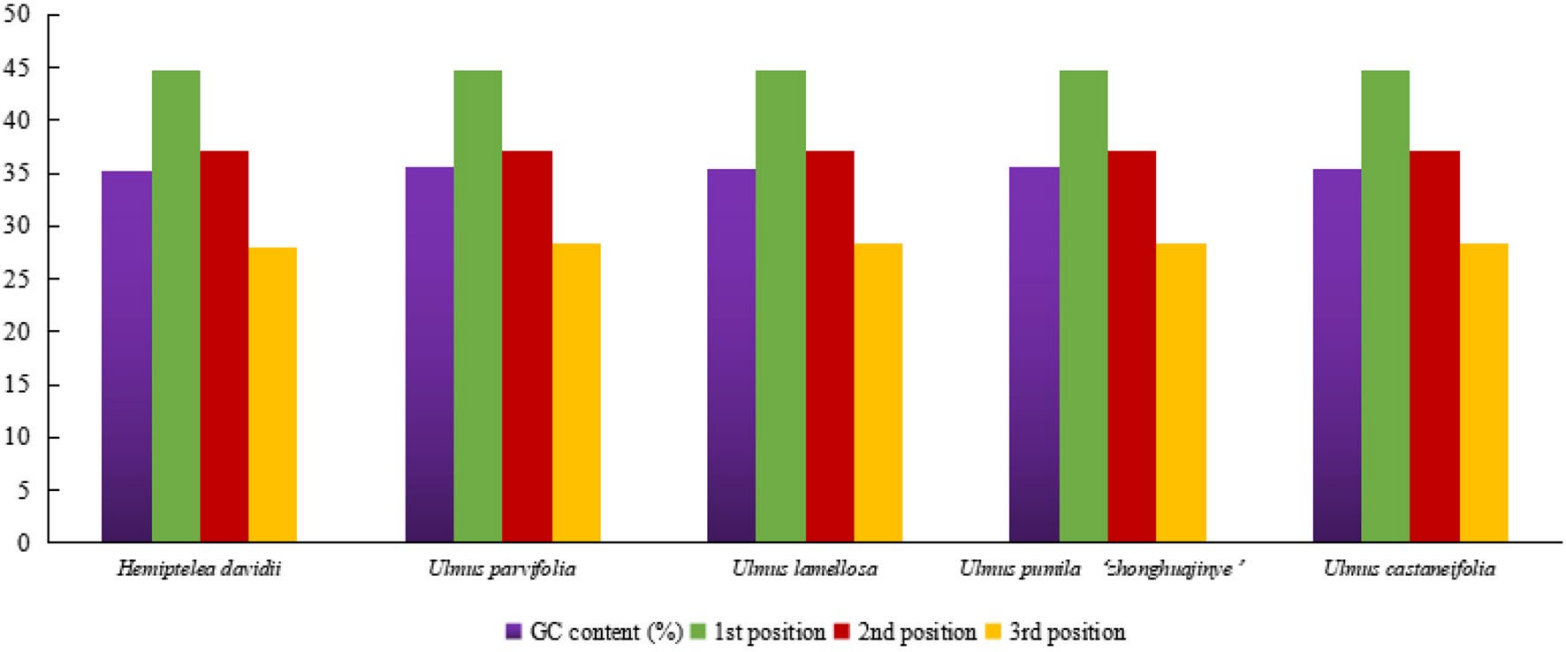
The GC (%) content in different positions of CDs region of species within four *Ulmus* species and *H. davidii*.

**Figure 4:**
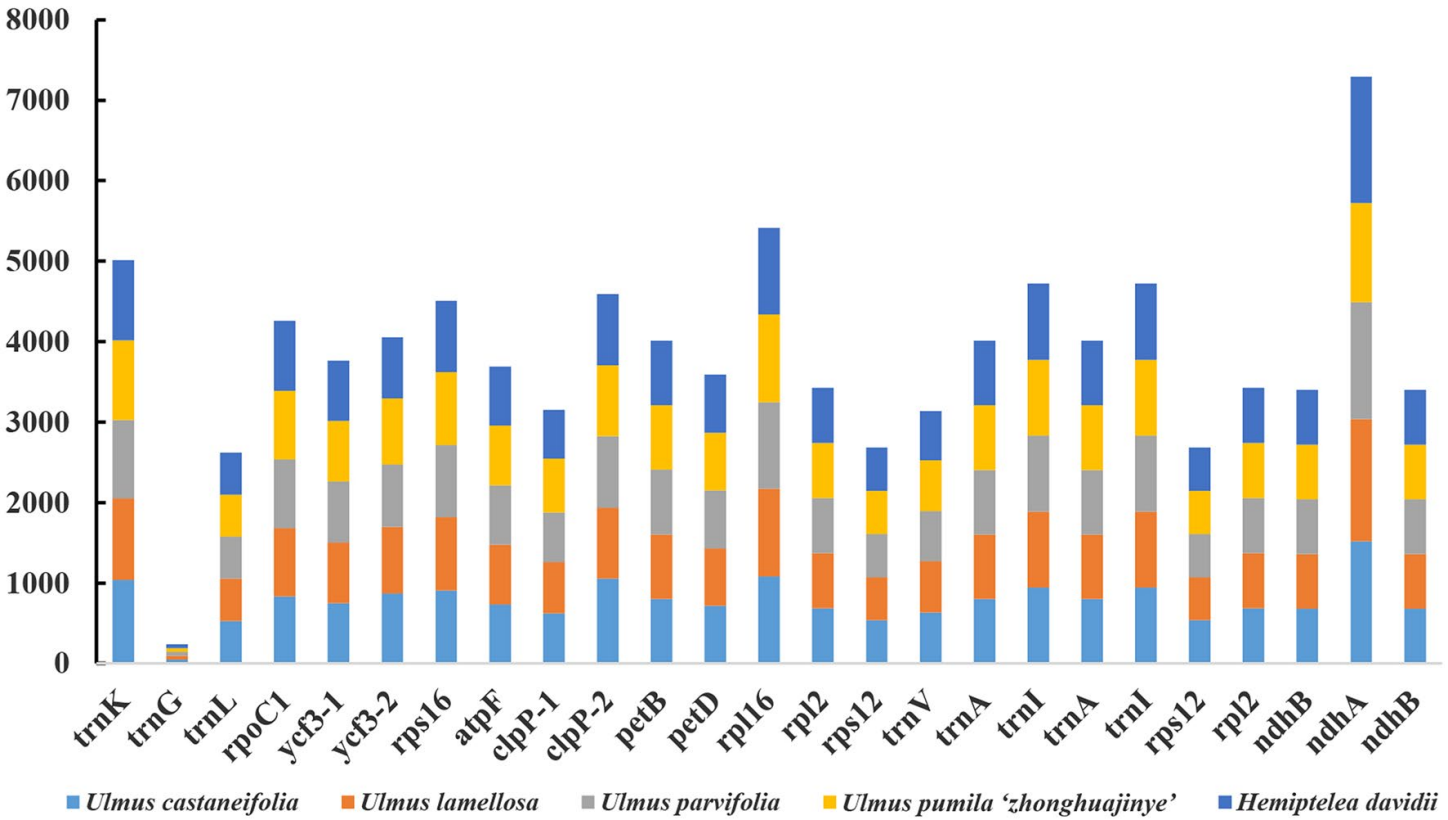
Intron length in the chloroplast genomes of four *Ulmus* species and *H. davidii*.

In addition, the total GC contents of the five species were similar, ranging from 35.30 to 35.62% which was higher than in the LSC and SSC regions, but lower than in the IR region. Moreover, the first position had the highest GC content than the second and third positions (Fig. 3). Comparative analysis indicated that gene structure was relatively conservative and most genes did not contain introns. In this study, the number of genes containing introns were 23. Among these, the *clpP* and *ycf3* genes contained two introns. The other genes contained only one intron that primarily involved 13 coding genes (*rps16*, *atpF*, *rpoC1*, *rpl2* × 2, *ndhB* × 2, *rps12* × 2, *ndhA, petB, petD* and *rpl16*) and eight tRNA genes (*trnK*, *trnG*, *trnL*, *trnV*, *trnI* × 2 and *trnA* × 2). The length of *ndhA* intron was the longest, followed by *rpl16* and *trnK* (Fig. 4).

### Gene loss and the Ka/Ks ratios of ulmaceae species pairwise

**Figure 5:**
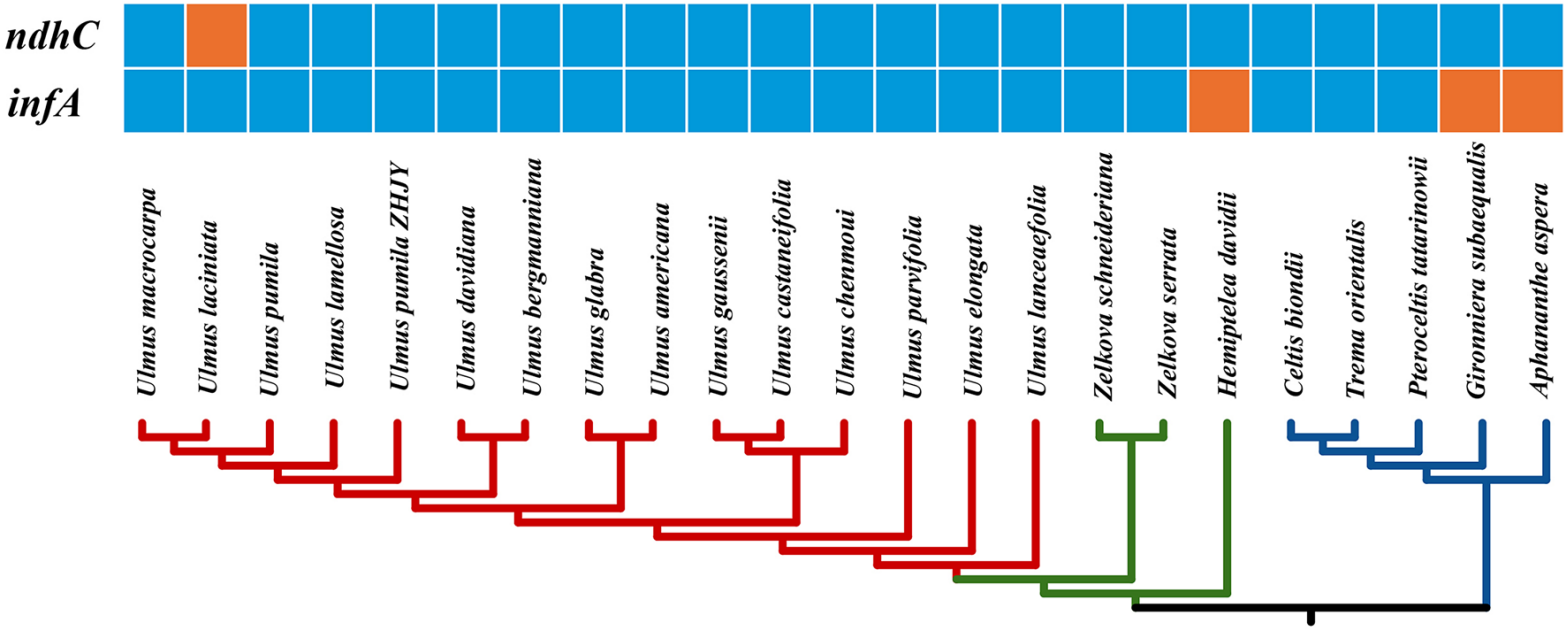
Loss of chloroplast protein-coding genes in the phylogeny of 23 Ulmaceae species.

The protein-coding genes of the 23 Ulmaceae species including 15 *Ulmus* species were counted. The results were shown in Fig. 5. As it was shown, the gene of *ndhC* was lost in *U. laciniata*. In addition, the *infA* was lost in three species (*H. davidii*, *G. subaequalis* and *A. aspera*) with different degree.

**Figure 6:**
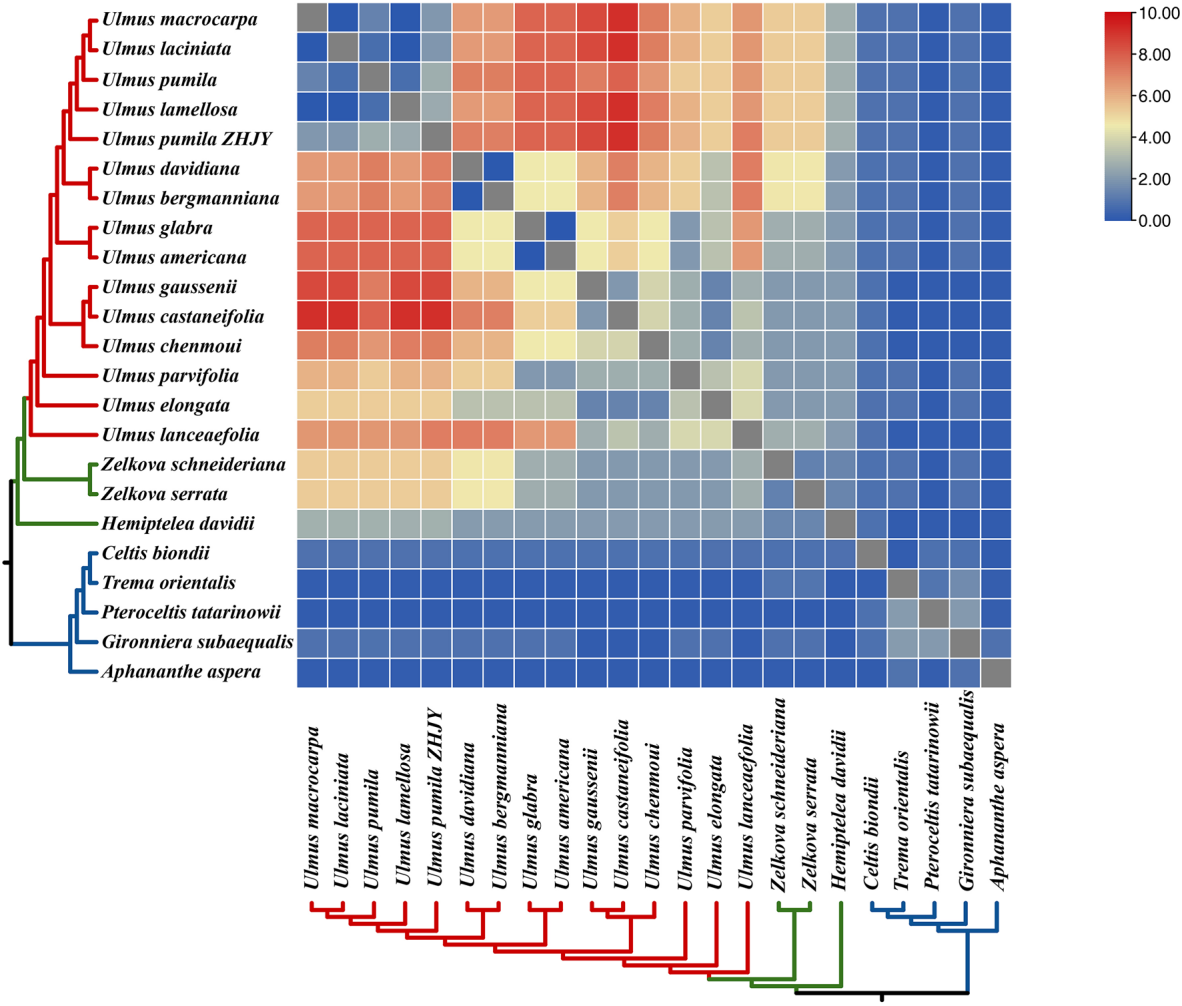
Pairwise Ka/Ks ratios in *Ulmus* and other Ulmaceae species.

The Ka/Ks ratios, which provided information on the effects of selection pressures on protein coding genes of each 23 Ulmaceae species pair, were calculated (Fig. 6). The results showed that the higher Ka/Ks ratios were detected in *Ulmus* species pairs than non-*Ulmus* species pairs.

### Positive selection analysis of protein sequence among Ulmaceae species

Seventy-seven common CDS genes from 23 Ulmaceae species were subjected to positive selection analysis (Table 2 and Supplementary Table S2). And Model A and Model A null were calculated using codeML. The results showed that no genes were positively selected. However, the BEB analysis indicated that two protein-coding genes (*rps15* and *rbcL*) had significant posterior probabilities and there was a positive selection site in each gene. Besides the *rps15* and *rbcL* genes were located in the SC region.

**Table 2:** The potential positive selection test based on the branch-site model.

No	Genes	Null hypothesis			Alternative hypothesis			Significance test	
		lnL	df	omega (w = 1)	lnL	df	omega (w > 1)	BEB	LRT/P-value
1	*psbC*	− 2628.876655	48	1	− 2628.876655	49	2.38616		1
2	*psbD*	− 1886.150227	48	1	− 1886.150206	49	1		0.99482915
3	*psbE*	− 438.426396	48	1	− 438.426396	49	1		1
4	*psbF*	− 207.535187	48	1	− 207.535187	49	2.13411		1
5	*psbH*	− 461.853033	48	1	− 461.853033	49	1		1
6	*psbI*	− 198.463720	48	1	− 198.463720	49	1.02032		1
7	*psbJ*	− 268.641875	48	1	− 268.641875	49	1		1
8	*psbK*	− 385.952605	48	1	− 385.952606	49	2.30325		1
9	*psbL*	− 184.308851	48	1	− 184.308851	49	2.85599		1
10	*psbM*	− 158.730809	48	1	− 158.730809	49	1.99465		1
11	*psbN*	− 226.751297	48	1	− 226.751297	49	2.31959		1
12	*psbT*	− 182.788331	48	1	− 182.788328	49	1		0.99804559
13	*psbZ*	− 323.223609	48	1	− 323.223610	49	2.04743		1
14	*rbcL*	− 2862.940558	48	1	− 2862.940558	49	1	97 F 0.589	1
15	*rpl14*	− 740.033667	48	1	− 740.033667	49	1.87798		1
16	*rpl16*	− 817.516599	48	1	− 817.516578	49	1		0.99482915
17	*rpl20*	− 784.139114	48	1	− 784.139113	49	1.63776		0.99887162
18	*rpl22*	− 1192.404192	48	1	− 1192.404192	49	1		1
19	*rpl23*	− 489.910969	48	1	− 489.910972	49	1.60907		1
20	*rpl2*	− 1318.425181	48	1	− 1318.425181	49	2.00600		1
21	*rpl32*	− 378.674247	48	1	− 378.674247	49	1		1
22	*rpl33*	− 408.488673	48	1	− 408.488673	49	1.59913		1
23	*rpl36*	− 172.345096	48	1	− 172.345096	49	1.67091		1
24	*rpoA*	− 2432.015315	48	1	− 2432.015306	49	1		0.99661487
25	*rpoB*	− 6520.420040	48	1	− 6520.420227	49	2.26257		1
26	*rpoC1*	− 4522.103881	48	1	− 4522.103903	49	1.78582		1
27	*rpoC2*	− 10,541.83165	48	1	− 10,541.83152	49	2.51647		0.98689001
28	*rps11*	− 832.170672	48	1	− 832.170681	49	1.72878		1
29	*rps12*	− 638.263354	48	1	− 638.263335	49	13.88404		0.99508154
30	*rps14*	− 610.532984	48	1	− 610.532987	49	1.52180		1
31	*rps15*	− 719.532818	48	1	− 719.270557	49	18.60132	30 D 0.873	0.46891907
32	*rps16*	− 605.186225	48	1	− 605.186223	49	1		0.99840423

### Repeat sequence analysis of Ulmaceae species

**Figure 7:**
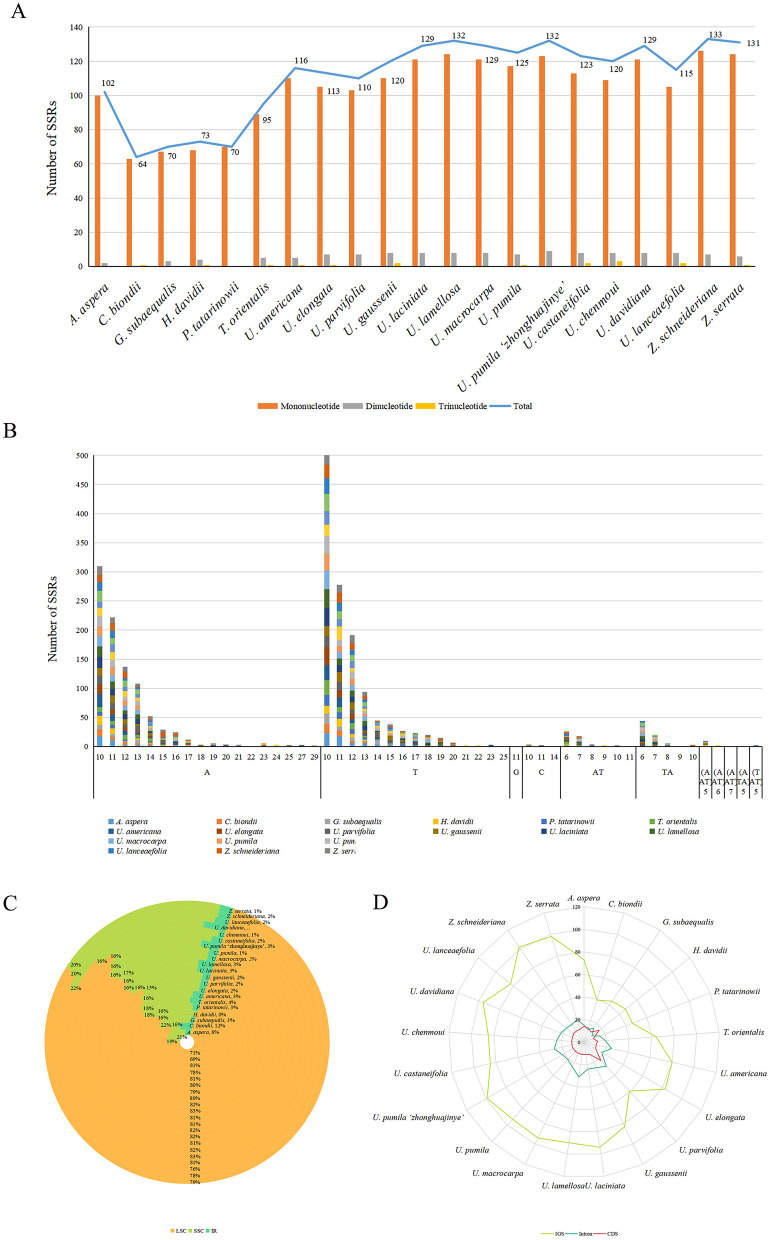
(**A**) Analysis of SSRs in the cp genomes of 21 Ulmaceae species; (**B**) The numbers of SSRs types of 21 Ulmaceae species; (**C**) Frequency of SSRs in the LSC, IR and SSC region; (**D**) Frequency of SSRs in intergenic regions, protein-coding genes and introns regions.

A total of 64–133 SSRs were identified in the cp genome of the 21 Ulmaceae species, with lengths of 10–29 bp, including mononucleotides, dinucleotides and trinucleotides. The mononucleotide repeats ranged from 63 to 126, followed by dinucleotide (2–9) and trinucleotide (1–3) repeats (Fig. 7A). The mononucleotides repeats were mostly composed of (A)_n_ and (T)_n_, with only one (G)_11_-type SSR in *G. subaequalis*; one (G)_10_-type SSR in *P. tatarinowii*, *T. orientalis*, *U. elongata* and *U. pumila* ‘zhonghuajinye’; one (C)_11_-type SSR in *A. aspera* and *U. parvifolia*; and one (C)_14_-type SSR in *U. lanceaefolia*. Dinucleotide repeats included 11 SSRs of (AT)_n_ and (TA)_n_ of different lengths. Besides, trinucleotide repeats included (AAT)_n_, (ATA)_5_ and (TAT)_5_ SSRs of different lengths (Fig. 7B).

The statistical results for the SSR distribution in the LSC, SSC and IR regions of the cp genome indicated that the SSRs in the 21 Ulmaceae species were mainly distributed in the LSC region with 44–107 SSRs, accounting for 69–83% of the total; followed by the SSC region with 11–27 SSRs, accounting for 15–22%; and the IR region with 0–8 SSRs, accounting for 0–12%. SSRs in *H. davidii* were only distributed in the LSC and SSC regions (Fig. 7C). In addition, SSRs were primarily distributed in intergenic regions ranging from 39 to 102 SSRs, while 9–31 occurred in introns and 9–22 occurred in CDS (Fig. 7D).

In the 21 Ulmaceae species, palindrome repeats (P), forward repeats (F), reverse repeats (R) and complement repeats (C) of repeat sequences were observed. *C. biondii* was the only species that lacked C repeats. The total number of repeat sequences ranged from 46 to 89 (21–35 of type P, 17–41 of type F, 1–17 of type R and 0–11 of type C), with *G. subaequalis* containing the fewest and *U. gaussenii* and *U. chenmoui* containing the most number (Fig. S1A). Moreover, the lengths of repeats primarily ranged from 30 to 39 bp, although three repeats were longer than 200 bp in *U. americana*, *U. gaussenii* and *U. castaneifolia* (Fig. S1B).

### Chloroplast genomic divergence and hotspots regions

**Figure 8:**
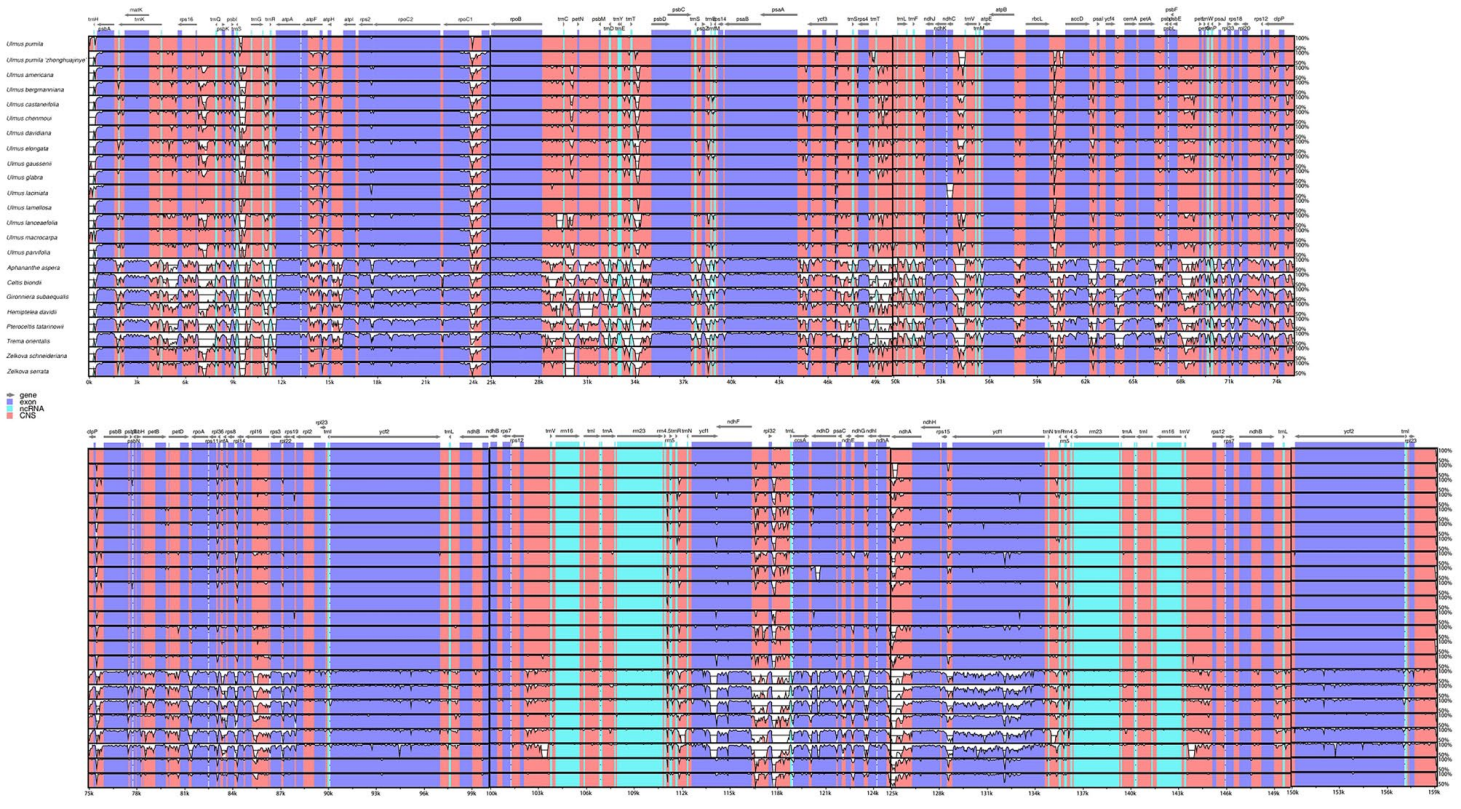
Sequence identity plots of the 23 Ulmaceae species genomes generated using mVISTA, taking the annotation of *U. pumila* as a reference. Grey arrows above the alignment indicate the orientation of genes. Blue bars represent exons, pink ones represent non-coding sequences (CNS). X-scale axis represents the genome coordinate positions, Y-scale axis represents the percent identity within 50–100%.

The mVISTA was used to compare and analyze the divergent regions of plastomes among the 23 Ulmaceae species with *U. pumila* as a reference. (Fig. 8). Overall, the 23 Ulmaceae species could be roughly divided into two groups: one containing 15 *Ulmus* species and two *Zelkova* species species; the other containing *H. davidii*, *A. aspera*, *C. biondii*, *G. subaequalis*, *P. tatarinowii* and *T. orientalis*. Significant separation was observed between the two groups. And the results showed that the cp genomes of *Ulmus*, *Zelkova* and *Hemiptelea* species were more conserved than the species of other group. In terms of region variation, the variation range of the LSC and SSC regions were greater than that of the IR regions. Moreover, the conservation of gene-coding regions was generally higher than that of non-coding regions. For example, the non-coding regions of *trnH/psbA, trnK/rps16* and *trnS/trnG* exhibited large variation and could be used as an alternative region for DNA barcoding at later stages. Although the gene-coding region was overall highly conserved, the conservativeness of the *ycf1* and *ndhD* genes was poor. These noncoding region and gene-coding region obtained could also be used as alternative regions for DNA barcoding of *Ulmus* and Ulmaceae species.

**Figure 9:**
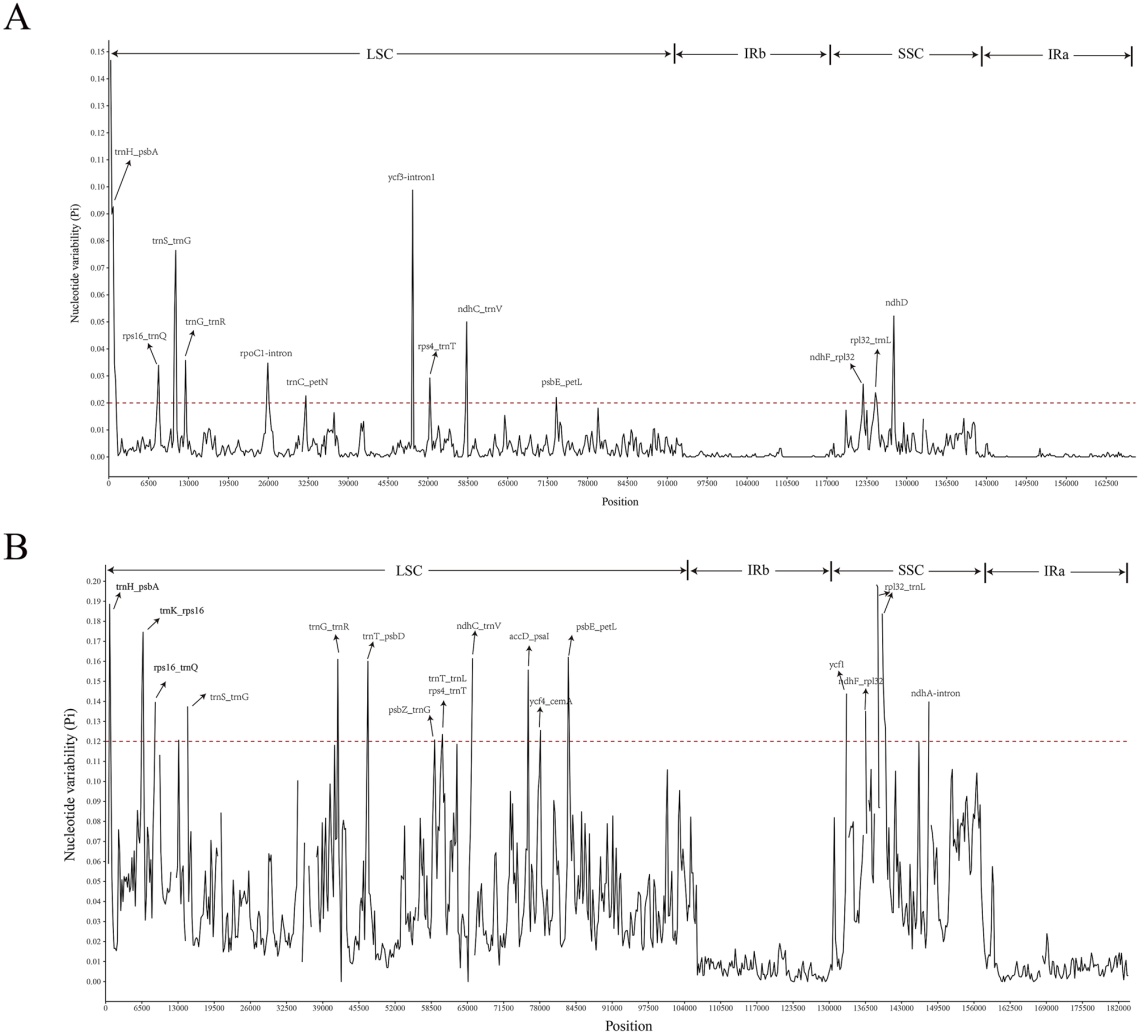
Nucleotide variability (pi) values of 15 *Umlus* species and 23 Ulmaceae species. (**A**): pi values of 15 *Umlus* species. (**B**): pi values of 23 Ulmaceae species. X-axis: name of the regions; Y-axis: nucleotide diversity. window length: 300 bp; step size: 200 bp.

To further clarify the diversity of *Ulmus* and Ulmaceae species at the sequence level, the nucleotide difference (pi) of the 15 *Ulmus* species and 23 Ulmaceae species were calculated respectively and suitable polymorphic loci from protein-coding sequences, IGS regions and intronic regions were identifed. The results showed that the most of the regions with the high nucleotide diversity among 15 *Ulmus* species were included from IGS regions, namely *trnH/psbA*, *rps16/trnQ*, *trnS/trnG*, *trnG/trnR*, *rpoC1-intron*, *trnC/petN*, *ycf3-intron1*, *rps4/trnT*, *ndhC/trnV*, *psbE/petL*, *ndhF/rpl32*, *rpl32/trnL*. The protein-coding regions of *ndhD* were also included in the suitable polymorphic loci (Fig. 9A, Table 3)*.* What is more, these variation locis were mainly distributed in LSC and SSC region.

**Table 3:** High variable marker of cp genomes among 15 *Ulmus* species.

High/variable/marker	Variable sites	Parsimony informative sites	Nucleotide diversity
*trnH/psbA*	106	97	0.1469388
*rps16/trnQ*	34	34	0.0339336
*trnS/trnG*	85	75	0.0764813
*trnG/trnR*	11	10	0.0357410
*rpoC1-intron*	43	40	0.0347718
*trnC/petN*	19	17	0.0226560
*ycf3-intron1*	6	5	0.0988095
*rps4/trnT*	34	34	0.0292303
*ndhC/trnV*	60	60	0.0500000
*psbE/petL*	22	19	0.0220286
*ndhF/rpl32*	18	17	0.0269951
*rpl32/trnL*	17	17	0.0237776
*ndhD*	72	72	0.0521942

In addition, We also compared all the regions of cp genomes of the 23 Ulmaceae species in pairwise alignment. the cp genome variation primarily occurred in intergenic regions (Fig. 9B, Table 4), such as *trnH/psbA, trnK/rps16, rps16/trnQ, trnS/trnG, trnG/trnR, trnT/psbD, psbZ/trnG, rps4/trnT, trnT/trnL, ndhC/trnV, accD/psaI, ycf4/cemA, psbE/petL, ndhF/rpl32, rpl32/trnL* and *ndhA-intron.* In the coding regions, the most variable gene was *ycf1* which showing that the gene-coding regions were more conservative than the non-coding regions. Thus, these region could be used as a potential molecular marker for the identification and phylogenetic analysis of *Ulmus* and Ulmaceae species.

**Table 4:** High variable marker of cp genomes among 23 Ulmaceae species.

High variable marker	Variable/sites	Parsimony/informative/sites	Nucleotide/diversity
*trnH/psbA*	63	47	0.1885375
*trnK/rps16*	55	45	0.1745718
*rps16/trnQ*	32	27	0.1396574
*trnS/trnG*	31	24	0.1206239
*trnG/trnR*	28	24	0.1374807
*trnT/psbD*	32	22	0.1609912
*psbZ/trnG*	26	23	0.1600226
*rps4/trnT*	24	20	0.1207277
*trnT/trnL*	52	35	0.1235178
*ndhC/trnV*	36	28	0.1615283
*accD/psaI*	35	29	0.1556324
*ycf4/cemA*	14	14	0.1254941
*psbE/petL*	42	37	0.1620553
*ycf1*	32	31	0.1438735
*ndhF/rpl32*	16	15	0.1351779
*rpl32/trnL*	122	88	0.1982872
*ndhA*-intron	62	49	0.1398927

### Phylogenetic analysis of Ulmaceae species

**Figure 10:**
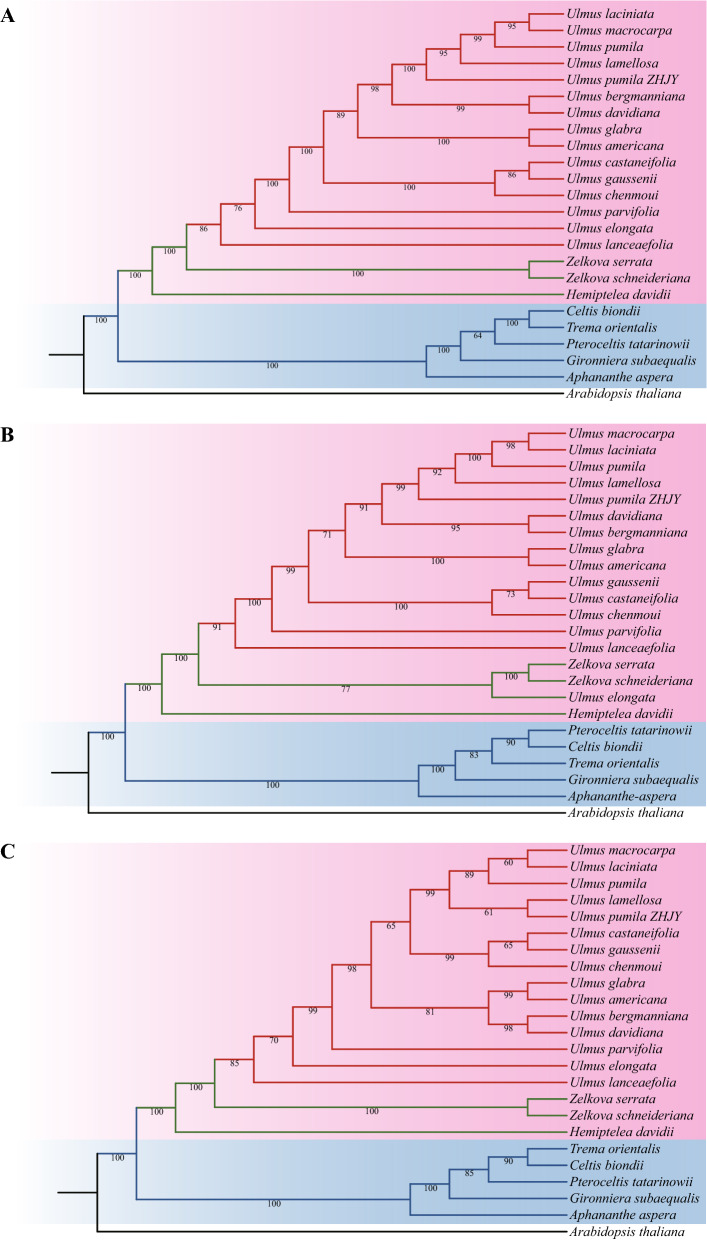
Phylogenetic trees of 23 Ulmaceae species. (**A**) Phylogenetic tree constructed using common protein coding genes; (**B**) Phylogenetic tree constructed using the whole cp genome; (**C**) Phylogenetic tree constructed using common genes in IR region.

To reveal the developmental relationship of Ulmaceae species, the phylogenetic tree based on the whole cp genome sequences, common protein-coding genes and common genes in IR region of 23 Ulmaceae species were constructed using the ML method. The results of three phylogenetic trees were nearly similar to a certain extent (Fig. 10). The 23 Ulmaceae species could be divided into two branches: one included *Ulmus*, *Zelkova* and *Hemiptelea*, among which *Hemiptelea* was the first to differentiate; and the other included *Celtis*, *Trema*, *Pteroceltis*, *Gironniera* and *Aphananthe*. Of the three trees, the one based on the whole cp genome and the common protein genes were more similar, and the *U. lanceaefolia* and *U. elongata* had the different locations. *U. lanceaefolia* was differentiated after *Zelkova* in Fig. 10A, while in Fig. 10B the *U. lanceaefolia* was differentiated after *Zelkova* and *U. elongata*. Besides the genetic relationship between *C. biondii*, *T. orientalis*, *P. tatarinowii* were different. The phylogenetic relationship of *Ulmus* species constucted based on IR region was different from the above two methods (Fig. 10C). For example, the *U. chenmoui* had a more closer relationship with *U. glabra and U. americana.*

## Discussion

### Cp genome variation of Ulmaceae species

In the present study, the cp genome size, structure and composition of the four *Ulmus* species and *H. davidii* were highly conserved, displaying a typical quadripartite structure with a LSC, a SSC region and two IR regions, which was similar to the other angiosperms^48^. The cp genome of the five species ranged from 159,113 to 160,388 bp, encoding 130–131 genes, including 85–86 protein coding genes, 37 tRNAs and eight rRNAs. In particular, *rps12* in Ulmaceae was recognized as the trans-spliced gene, which was in consistent with observations in other species^49^_._ The five species shared the similar GC content (about 35%). Besides, the overall difference in cp genome size was 1275 bp and the difference in LSC length was 730 bp, accounting for the majority of the cp genome variation. Therefore, the differences in cp genome length of the five species were primarily caused by variation in LSC length based on IR contraction or expansion^50^. In this study, the gene introns of the five species were compared and analyzed and the results indicated that most genes do not contain introns. There were only 23 genes harbored introns and no intron loss was found in the five species. Among them the *clpP* and *ycf3* gene contained two introns, which is similar with the other plants^51^. Intron sequences were valuable in phylogenetic studies at lower taxonomic levels (e.g., closely related genera and interspecies)^52^. Huang et al.^53^ analyzed the phylogenetic relationship of the four species among *Amana* by combining partial DNA fragments of ITS nuclear sequence and *trnL intron* sequence, and proved that *Amana wanzhensis* was an effective species. Moreover, Huang et al.^54^ confirmed that the intron of the *ndhA* gene was a promising DNA barcode for *Fagopyrum* phylogenetic research. In this study, the *ndhA* (1233–1570 bp) gene had the longest introns. And the length of the *ndhA* gene intron varied the 337 bp among the five species. In the future, the intron of the *ndhA* gene may similarly used as a DNA barcode for the phylogenetic study of *Ulmus*, which will serve to facilitate the identification and utilization of natural *Ulmus* resources. The phenomenon of gene loss was common in most plant^55^. In the present study, the *infA* and *ndhC* gene were lost in different species, which was also occured in previous reported ^56^.

### Identification of repeated sequences among Ulmaceae species

cpSSRs, which are uniparentally inherited materia and widely distribute in the genome of eukaryotes, with the characteristics of simple structures, small molecular weight and relative conservation, are short tandem repeats of 2 to 6 bp and widely used in species identification, genetic difference analysis at the individual level and population evolution studies^57,58^. In this study, a total of 64–133 SSRs were found in the cp genomes of 21 Ulmaceae species, including mononucleotide, dinucleotide and trinucleotide types. The numbers of mononucleotides were the largest among all the types and contributed to AT richness, which was similar to previous results^59,60^. The distribution of SSR loci in different regions was uneven and primarily occurred in the LSC region, SSC region and intergenic region, and less so in the IR region, gene region and introns. In addition, previous studies had reported that new genes had been generated from repetitive sequences, and SSR loci were more distributed in SCs, which may be one reason for their greater variation compared to the IR region^61^.

### Adaptative evolution of the Ulmaceae plastome

In CodeML, there were four common models including branch model, site model, branch-site model and clade model. Among them, the branch-site model was usually used to assess potential positive selection of genes, in which the nonsynonymous and synonymous rate ratio (ω = dN/dS) was used to measured selection pressure and the ratio ω < 1, ω = 1, ω > 1 were considered to be purifying selection, neutral selection and positive selection, respectively^62,63^. Then the BEB method was further used to assess whether sites were under positive selection^64^. The analysis of adaptive evolution of genes is of certain value for studying the changes of gene structure, gene function, and evolutionary track of species^65^. The plastid genes with positive selection signature suggested that in response to the environment these genes might be undergoing adaptative evolution^66^. The cp genome was highly conserved and few genes with positive selection were identified, which is consistent with other studies^67^. For example, it was found that the *rpoB, matk*, *ndhF, rps18, rps7, ycf4, clpP* and *rbcL* genes were positively selected^61^. And the *rpoB* and *matK* gene has been used as DNA barcodes in phylogeny reconstruction of plants^68,69^. In this study, the positive selection analysis of 77 protein-coding genes among 23 Ulmaceae species indicated that there was no positively selected gene but the *rps15* and *rbcL* had positive selection sites, which is consistent with the study of Xie et al.^70^, in which there were no significant *p-*values, while, some genes like *petA*, *rps4*, *ndhE* and *rpoC1* were found with positive selection sites in the BEB test. The *rps15* was different types of small subunit ribosomal structural proteins. In addition to playing an important regulatory role in ribosomal biosynthesis, the gene were also involved in regulating a variety of cellular life processes, such as genome integrity and development^71–73^. Besides, the *rbcL* gene (large subunit of ribose-1,5-diphosphate) was located in the large region outside the reverse repeat sequence, which encoded the large subunit of Rubisco. Eight *rbcL* and eight *rbcS* genes encoded by nuclear genes constitute Rubisco, which mainly catalyzed the fixation of carbon dioxide during photosynthesis and the oxidation of carbon during photorespiration. The sequence of the *rbcL* gene had been widely used in molecular systematics research to detect the systematic relationship and molecular evolution between plants^74,75^. Wu et al.^76^ used a single fragment of *rbcL* to obtain a phylogenetic tree of mangrove plant with a higher average node support rate than the *matK* and *trnH-psbA* fragments, which could accurately distinguish different tree species.

### Identification of hotspots

DNA barcoding has been widely used in species identification, resource classification and phylogenetic evolution^77^. Cp genome thus plays an important role in the development of DNA barcoding. For example, the highly variable loci identified through sliding window and mVIST analysis in cp genome could be used as candidate markers for molecular markers, DNA barcoding and evolutionary analysis. Among them the molecular evolution rate of coding region and non-coding region is different, which is suitable for the phylogenetic study of different order. The coding region is suitable for the phylogenetic research of families, orders and even higher taxonomic levels, while the non-coding region is suitable for the phylogenetic research of genera and species^78^. For example, a phylogenetic tree based on the combined sequences of *trnL-trnF* and *accD-psaI* in the chloroplast noncoding region further confirmed the independent evolution of Eastern pear and Western pear from the maternal evolutionary background^79^_._ The *matK* gene, which exhibited rapid evolution and high polymorphism, was widely used as an important marker gene in evolutionary research and species identification^80^. Moreover, The regions such as *matK*, *rbcL* and *trnK/rps16* have been proved to be commonly used as DNA barcodes in plant identification^81^. In this study, the result of alignment and nucleotide diversity revealed the sequencing five species had high level of similarity. It is similar to the other species that the LSC and SSC regions were more variable than the IR regions, whereas the coding regions were more conservative than the non-coding regions^82^. Some polymorphic regions by comparison of 15 *Ulmus* species were also identified using the sliding window and mvista analysis. The most divergent regions were *trnH/psbA, rps16/trnQ, trnS/trnG, trnG/trnR, rpoC1-intron, trnC/petN, ycf3-intron1, rps4/trnT, ndhC/trnV, psbE/petL, ndhF/rpl32, rpl32/trnL* and protein-coding gene *ndhD*. Among them, *trnH-GUG/psbA*, *trnS/trnG* and *ndhF/rpl32* had already been screened as a suitable barcode for plants^83–85^. The *trnH/psbA* is widely used as a phylogenetic marker in the Asteraceae family^86^. These hotspot regions obtained in our study could be used as DNA border in plant identification and system evolution in *Ulmus* species.

### Phylogenetic analysis

The base substitution rate in the maternally inherited cp genome was much lower than that in the nuclear genome. Therefore, the cp genome had become an important basis for phylogenetic analysis of higher plants. In the Flora Reipublicae Popularis Sinicae (FRPS), *Ulmus* species were divided into four sections: Blepharocarpa, Chaetoptelea, Microptelea, and Ulmus. Section Ulmus was further divided into three series: Glabrae, Lanceaefoliae, and Nitentes. Among the five species sequenced in this study, *U. parvifolia* belongs to Sect. Microptelea; *U. castaneifolia* belongs to Ser. Nitentes of Sect. Ulmus; *U. lamellosa* and *U. pumila* ‘zhonghuajinye’ belong to Ser. Glabrae of Sect. Ulmus, and *H. davidii* is the only species of *Hemiptelea*, which is consistent with the results of constructing evolutionary trees from the cp genomes of 23 species. However, several differences existed. First, *U. lanceaefolia* belongs to Series Lanceaefoliae of Section Ulmus in the FRPS, but our results indicated that it did not belong to Section Ulmus. This discrepancy may be due to the fact that *U. lanceaefolia* was an evergreen plant, unlike other Ulmus species. A large amount of intraspecific variation in photosynthetic genes and intergenic regions of chloroplast genomes had been reported for other evergreen species^87^, leading to differences in evolutionary relationships. The second discrepancy was that *U. gaussenii* belongs to Series Glabrae of Section Ulmus in the FRPS, but our results indicated that this species was clustered into a small branch with *U. castaneifolia* and *U. chenmoui* of Series Nitentes. This result was consistent with classifications of *Ulmus* species based on leaf morphology, wood anatomical structure, and pollen morphology^88–90^. Based on the results of this study, *U. lanceaefolia* could be listed as a new *Ulmus* section or as a new genus of Ulmaceae in parallel with *Zelkova* and *Hemiptelea*. Furthermore, *U. gaussenii* could be included in Series Nitentes. However, the cp genome may not contain enough genetic information to thoroughly analyze the evolutionary relationship of Ulmaceae species; therefore, it is necessary to use nuclear genome information for further classification research.

## Supplementary Information

Supplementary Information.

## Data Availability

The original contributions presented in the study are publicly available. This data can be found at NCBI (MZ292512, MZ292513, MZ292514, MZ292515).
