# Supplementary material for: Complete chloroplast genome structure of four Ulmus species and Hemiptelea davidii and comparative analysis within Ulmaceae species
Source: Sci Rep. 2022 Sep 24;12:15953. doi: 10.1038/s41598-022-20184-w (PMC9509344; doi:10.1038/s41598-022-20184-w)
Supplement: Supplementary file 1 — Supplementary Information. [file 41598_2022_20184_MOESM1_ESM.pdf]

Table S1 species information

Number	Species	Family	Genus	Accession Number
1	*Ulmus parvifolia*	Ulmaceae	*Ulmus*	This study
2	*Ulmus lamellosa*	Ulmaceae	*Ulmus*	This study
3	*Ulmus pumila 'zhonghuajinye'*	Ulmaceae	*Ulmus*	This study
4	*Ulmus castaneifolia*	Ulmaceae	*Ulmus*	This study
5	*Hemiptelea davidii*	Ulmaceae	*Hemiptelea*	This study
6	*Ulmus glabra*	Ulmaceae	*Ulmus*	Unpublished
7	*Ulmus bergmanniana*	Ulmaceae	*Ulmus*	Unpublished
8	*Ulmus pumila*	Ulmaceae	*Ulmus*	NC_032721
9	*Ulmus americana*	Ulmaceae	*Ulmus*	NC_044473
10	*Ulmus elongate*	Ulmaceae	*Ulmus*	NC_046061
11	*Ulmus gaussenii*	Ulmaceae	*Ulmus*	NC_037840
12	*Ulmus laciniata*	Ulmaceae	*Ulmus*	NC_032719
13	*Ulmus macrocarpa*	Ulmaceae	*Ulmus*	NC_032720
14	*Ulmus chenmoui*	Ulmaceae	*Ulmus*	NC_037758
15	*Ulmus davidiana*	Ulmaceae	*Ulmus*	NC_032718
16	*Ulmus lanceaefolia*	Ulmaceae	*Ulmus*	NC_058620
17	*Aphananthe aspera*	Ulmaceae	*Aphananthe*	NC_039726
18	*Celtis biondii*	Ulmaceae	*Celtis*	NC_039727
19	*Gironniera subaequalis*	Ulmaceae	*Gironniera*	NC_039729
20	*Pteroceltis tatarinowii*	Ulmaceae	*Pteroceltis*	NC_039733
21	*Trema orientalis*	Ulmaceae	*Trema*	NC_039734
22	*Zelkova schneideriana*	Ulmaceae	*Zelkova*	NC_041074
23	*Zelkova serrata*	Ulmaceae	*Zelkova*	NC_040958

Table S2 The potential positive selection test based on the branch-site model

No.	Genes	Null hypothesis			Alternative hypothesis			Significance test	
		lnL	df	omega (w = 1)	lnL	df	omega (w > 1)	BEB	LRT_P-value
1	*accD*	-3874.117971	48	1	-3874.117952	49	1		0.99508154
2	*atpA*	-3044.791429	48	1	-3044.724017	49	1		0.713481864
3	*atpB*	-2864.816249	48	1	-2864.81626	49	2.46714		1
4	*atpE*	-844.361015	48	1	-844.361015	49	1.98765		1
5	*atpF*	-1356.634249	48	1	-1356.634251	49	1.69819		1
6	*atpH*	-475.6739	48	1	-475.673884	49	1		0.995486507
7	*atpI*	-1473.108736	48	1	-1473.108743	49	1.60162		1
8	*ccsA*	-2544.054903	48	1	-2544.054909	49	1		1
9	*cemA*	-1686.140457	48	1	-1686.140477	49	2.37918		1
10	*clpP*	-1252.029962	48	1	-1252.029962	49	1		1
11	*matK*	-4483.327829	48	1	-4483.327747	49	1		0.989782371
12	*ndhA*	-2387.920139	48	1	-2387.920145	49	2.34564		1
13	*ndhB*	-2137.375866	48	1	-2137.37587	49	2.04795		1
14	*ndhD*	-4236.652012	48	1	-4236.65216	49	5.60728		1
15	*ndhE*	-586.649861	48	1	-586.649861	49	1		1
16	*ndhF*	-6427.359057	48	1	-6427.359057	49	1		1
17	*ndhG*	-1336.095405	48	1	-1336.095396	49	1		0.996614873
18	*ndhH*	-2575.267307	48	1	-2575.267323	49	1.51179		1
19	*ndhI*	-1074.541696	48	1	-1074.541697	49	1.97447		1
20	*ndhJ*	-983.505982	48	1	-983.505986	49	1.63901		1
21	*ndhK*	-1502.513198	48	1	-1502.513198	49	1		1
22	*petA*	-2255.731352	48	1	-2255.731342	49	1		0.996431764
23	*petB*	-1233.618713	48	1	-1233.61869	49	1		0.994588525
24	*petD*	-943.679775	48	1	-943.679775	49	5.21947		1
25	*petG*	-198.330603	48	1	-198.330603	49	2.42736		1
26	*petL*	-198.375811	48	1	-198.375811	49	1		1
27	*petN*	-168.959407	48	1	-168.959407	49	1		1
28	*psaA*	-4169.025102	48	1	-4169.025137	49	2.29811		1
29	*psaB*	-4010.413208	48	1	-4010.413285	49	1.09444		1
30	*psaC*	-423.002257	48	1	-423.002257	49	2.02226		1
31	*psaI*	-211.093876	48	1	-211.093876	49	1		1
32	*psaJ*	-277.698808	48	1	-277.698808	49	1.6791		1
33	*psbA*	-2282.33528	48	1	-2282.335271	49	1		0.996614873
34	*psbB*	-2997.91126	48	1	-2997.911283	49	1		1

35	*rps19*	-412.644098	48	1	-412.644099	49	1	1
36	*rps2*	-1432.587016	48	1	-1432.587046	49	1.80093	1
37	*rps3*	-1643.233959	48	1	-1643.233967	49	2.26806	1
38	*rps4*	-1316.585727	48	1	-1316.585723	49	1.09787	0.997743245
39	*rps7*	-682.658149	48	1	-682.658149	49	2.21764	1
40	*rps8*	-1022.087003	48	1	-1022.087003	49	1.8015	1
41	*rps18*	-597.085368	48	1	-597.085367	49	2.264	0.998871621
42	*ycf1*	-19016.8542	48	1	-19016.85486	49	1	1
43	*ycf2*	-11470.53597	48	1	-11470.536	49	1.75119	1
44	*ycf3*	-1013.486751	48	1	-1013.486754	49	2.08366	1
45	*ycf4*	-1296.721459	48	1	-1295.122438	49	1	0.073726488

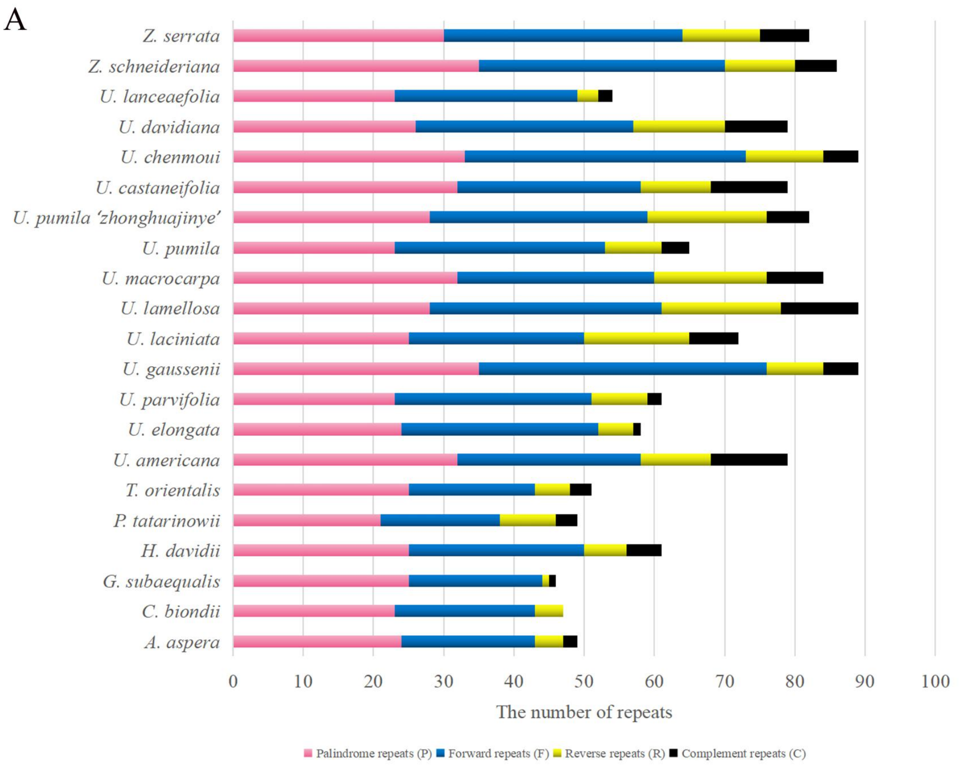

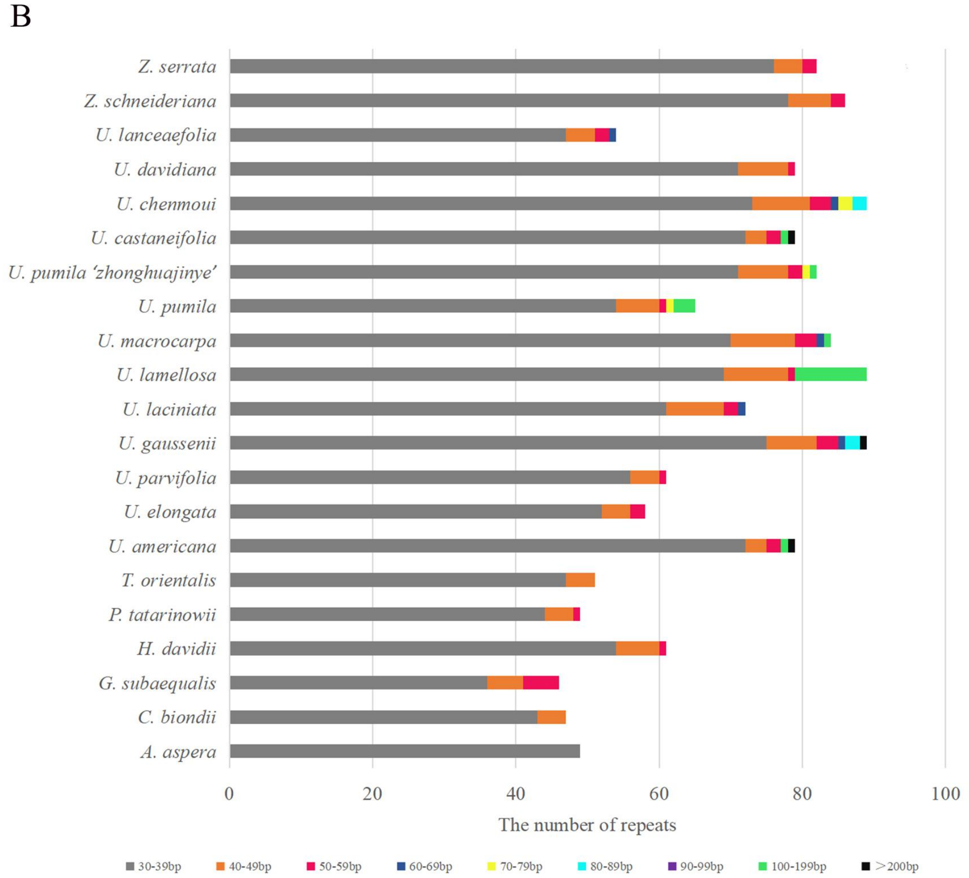

Figure S1. Analysis of repeats in 21 Ulmaceae species chloroplast genomes, (A) Number of Palindromic repeat, Direct repeat, Reverse repeat, Complement repeat; (B) Number of tandem repeats in different lengths.
